# Lumbar Spinal Cord Activity and Blood Biochemical Changes in Individuals With Diabetic Peripheral Neuropathy During Electrical Stimulation

**DOI:** 10.3389/fneur.2019.00222

**Published:** 2019-03-18

**Authors:** Yanlong Jia, Zhiwei Shen, Guisen Lin, Tingting Nie, Tao Zhang, Renhua Wu

**Affiliations:** ^1^Department of Medical Imaging, Second Affiliated Hospital, Shantou University Medical College, Shantou, China; ^2^Provincial Key Laboratory of Medical Molecular Imaging, Shantou University Medical College, Shantou, China

**Keywords:** spinal cord, functional magnetic resonance imaging, diabetes, peripheral neuropathy, electric stimulation

## Abstract

It is difficult to perform an *in vivo* evaluation of the nerve conduction mechanism in a patient with diabetic peripheral neuropathy (DPN). We aim to explore possible activation differences to enable a further understanding of the nerve conduction mechanisms of diabetic neuropathy and to present a novel clinical method to evaluate nerve injury and recovery. DPN patients (*n* = 20) and healthy volunteers (*n* = 20) were included in this study to detect the functional activation of the lumbar spinal cord via electric stimulation. Spinal fMRI data sets were acquired via a single-shot fast spin echo (SSFSE) sequence. A task-related fMRI was performed via low-frequency electrical stimulation. After post-processing, the active voxels and the percentage of signal changes were calculated for the DPN evaluation and the correlations between the blood biochemical indexes, such as glucose, total cholesterol, and hemoglobin A1c were explored. Activation in the DPN patients was primarily observed in the T12 (10/13) vertebral level. The percentage of signal changes in DPN patients was higher than that in the control group (Z = −2.757, *P* < 0.05). Positive correlation between the percentage of signal changes and the total cholesterol/glucose in the DNP group was found (*P* < 0.05). Lumbar spinal cord fMRI, based on the SEEP effect, was determined to be feasible. The repetitive activation distribution was primarily located at the T12 vertebral level. Lumbar spinal cord fMRI might be used as a potential tool to assess and reveal the nerve conduction mechanisms in DPN.

## Introduction

Peripheral neuropathy is a major complication of long-term diabetes. Sensory abnormalities are a common early symptom caused by diabetic peripheral neuropathy (DPN), the most common symptom being loss of sensation in the extremities. The pathogenesis of DPN remains unknown, difficult to objectively assess, and there are no treatment options to either prevent or delay the progression of diabetic neuropathy, other than early effective blood sugar control. It is well known that diabetic peripheral lesions are typically associated with metabolic abnormalities (1), such as glucose and lipid metabolism disorders. Glucose metabolism disorder is a widely recognized risk factor for DPN, and current evidence suggests that lipid metabolism disorder is a risk factor for macrovascular diseases (e.g., cardiovascular and cerebrovascular diseases). However, it is widely believed that a correlation exists between blood lipid levels and DPN caused by vascular dysfunction (microangiopathy), but this is yet to be proven. In addition, most studies (2, 3) have focused solely on DPN, and the involvement of the central nervous system has been largely overlooked. It is, therefore, particularly important to study the relationship between metabolic abnormalities and neuronal activity in DPN.

Animal models have been used to explore the potential mechanism of DPN, and the peripheral sensory neurons of diabetic rats exhibit many structural, functional, and neurochemical disorders (4–6). All these are considered to reflect early stages of progressive sensory loss and to distal degenerative neuropathy mostly seen in diabetic patients. However, when investigators applied behavioral sensorimotor tests in diabetic rats, the relationship between the sensory loss phenotype and the behavioral sensorimotor test was less consistent, some reports showing allodynia and hyperalgesia rather than hypoalgesia in diabetic rats (7, 8). Multiple interpretations have been provided for these phenomena. Some evidence suggested that diabetes induces the exaggerated activity of primary afferents (9, 10), whereas other studies indicated that the spinal cord and higher central nervous system (CNS) may be involved in sensory information processing, leading to the behavioral indicators of allodynia and hyperalgesia (11, 12). It is, therefore, of great interest to measure neuronal activity in DPN patients, to gain insight into potential sites of signal amplification.

Currently, the gold-standard method for diagnosing DPN remains electromyography (EMG). However, since the procedure is tedious and time-consuming, and characterized by poor specificity, many DPN patients are often not diagnosed at the early stages. Although molecular imaging modalities such as single photon emission computed tomography (SPECT), positron emission tomography (PET), and magnetic resonance spectroscopy (MRS) have been applied to explore the functional changes in DPN patients, these modalities are not suited to routine clinical use because of their high cost, the risks related to radiation, and lengthy scanning time. It is therefore necessary to detect the neuronal activation in diabetes patients via a noninvasive, radiation-free, and practical tool *in vivo* to determine the possible mechanisms of signal transduction.

Functional magnetic resonance imaging (fMRI), characterized by high spatial and temporal resolution, is used for noninvasive detection of neuron activity in the brain. Since its introduction in 1996, fMRI has also been used to study the spinal cord, based on the blood-oxygenation level dependent (BOLD) contrast with echo planar image sequence acquisition (13). Over the past two decades, obvious progress in spinal fMRI methods have been made (8, 14–16). Specific functional locations of activity in the lumbar spinal cord have been observed after leg motor or sensory stimulation (17–20), and the signal changes that are acquired by spinal fMRI are consistent with the nerve conduction theory. However, being subject to motion and local field inhomogeneities, over-dependence on field strength and echo time, spinal fMRI images acquired from gradient-echo sequence or echo planar image show low signal to noise ratio (SNR) and distortion (21). A new contrast mechanism based on the fast echo spin sequence acquisition is the signal enhancement from extra-vascular water protons (SEEP) (17, 22, 23), which present several advantages over the BOLD signal: insensitivity to magnetic field inhomogeneity, independence from field intensity and echo time, better spatial localization of neuronal activity, and a relatively higher SNR and tissue contrast-to-noise ratio (CNR). In addition, several studies have proven that SEEP contrast is an important dominant contrast mechanism in spinal cord fMRI fields (15, 22, 24, 25).

In this study we therefore used an optimized single-shot fast spin echo (SSFSE) protocol to detect neural signal changes and to map the activation location in the lumbar spinal cord of diabetes patients and healthy controls via electric stimulation. We aim to explore possible activation differences to enable further understanding of the nerve conduction mechanisms of DPN and to present a novel clinical method to evaluate nerve injury and recovery.

## Materials And Methods

### Subjects

Informed consent was obtained from all participants before enrollment in the study, which was reviewed and approved by the local Ethics Committee of the Second Affiliated Hospital of Shantou University Medical College. A total of 40 right-handed volunteers aged between 22 and 65 years (22 males and 18 females) were recruited and separated into two groups. The DPN group, included 20 patients with type-2 diabetes mellitus and DPN; these patients experienced loss of sensation of the extremities for 1–6 months (e.g., painless sensory symptoms, numbness, and weakness in lower limbs). The control group included 20 healthy volunteers. Participants were excluded from the study if any of the following conditions were met: (i) any contraindication for MRI (e.g., claustrophobia, cardiac pacemaker, or metal in the body); (ii) MRI revealed spinal cord injury, tumor, or myelitis; (iii) complaints of pain elsewhere in the body (e.g., ache of lower limb, infection, or backache); and (iv) any neurological disorder or muscle-related diseases diagnosed by two experienced neurologists and a radiologist, according to clinical symptoms and the MRI. Blood biochemistry information (glucose, total cholesterol, and hemoglobin A1c) of the DPN patients and controls (glucose only) was collected. The clinical symptoms and EMG were used to confirm the diagnosis of DPN. No EMG tests were performed on healthy volunteers.

### Electrical Stimulation Paradigm

Electrical stimulation was applied with a low-frequency electrical stimulator G9805-C (Shanghai, Medical Electronic Instrument Factory, intermittent pulse, frequency 20 Hz) according to clinic rehabilitation therapy protocols. The stimulation current was adjusted to 5–7 mA, giving the subjects a sense of swelling without being uncomfortable. Although DPN patients had painless sensory symptoms and numbness, they could still feel the electrical simulation when it was applied during the fMRI studies. The stimulation probe was 3 × 3 cm and was placed against the skin on the right anterolateral leg to stimulate the L4–L5 dermatome, whose spinal cord levels match the craniocaudal distance spanned by the caudal quarter of the T11 vertebral body and the rostra quarter of the L1 vertebral body. In each experiment, the first two volumes were discarded in order to reach steady-state during the time-series acquisition. Two hundred and twenty-two images were acquired with alternating blocks of four rest- and three activation periods of electrical stimulation. The total time required was 11 min 57 s.

### MR Data Acquisition

MR data acquisition was performed on a 1.5 Tesla clinical MR system (Signa HDX, General Electric) with an eight-channel phased-array spine coil for receiving MR signals. Anatomy images were acquired by a fast spin echo sequence with scanning parameters (sag/axial of T1-weighted): TR = 548/500 ms, TE = 10/16 ms, thickness = 4/7 mm, space = 0.5 mm, FOV = 24 × 24 cm, parent = 256 × 256, NEX = 2, and ETL = 32. Functional time-course data were obtained using a single-shot fast spin-echo sequence with parameters: TR = 6,000 ms, echo time of 6.6 ms, FOV = 24 × 24 cm, matrix = 256 × 256, NEX = 1, and ETL = 64. Six sagittal slices were set spanning the entire lumbar spinal cord with a thickness of 4 mm and a spacing of 0.5 mm. Six axial slices with a thickness of 7 mm and a space of 0.5 mm were obtained from the T12 vertebral level. Flow compensation gradients were used in the through-slice direction to reduce body motion artifacts during the electric stimulation. Phase-encoding in the right/left direction was chosen, so that motion artifacts from organs, such as the heart and lungs did not spread across the spinal cord in the resulting images. Four spatial saturation pulses were also positioned around the spine to eliminate the signal from regions outside the FOV, such as breathing and back movement.

### fMRI Data Analysis

fMRI data fseries' were pre-processed using MRIcro software (Chris Rorden, University of South Carolina, USA http://www.mccauslandcenter.sc.edu/mricro/), which generated a 3D data set within one TR time. Datasets were analyzed using Statistical Parametric Mapping (SPM8) software (Functional Imaging Laboratory, Wellcome Trust Center for Neuroimaging, Institute of Neurology, University College London, UK http://www.fil.ion.ucl.ac.uk/spm/software/spm8/) in MATLAB R2011b. The directions of the image plane were adjusted to be consistent with the three planes of the SPM8 default orientations. It is common for fMRI signals to be corrupted by random and physiological noise, so it is important to perform processing to remove these artifacts. The major steps involved motion correction, where a mean functional image was used as a template for the spatial standardization via SPM8 software. In this study, participants with a movement exceeding 2 mm of translation in the x, y, or z-axes or 2° of rotation around the three axes were excluded to minimize movement artifacts. The time-courses of the “active” and “rest” conditions were represented as a simple boxcar model and low-frequency components such as drift were removed. Individual level data were analyzed using a General Linear Model (GLM) for each run to compare the stimuli blocks to the rest periods. The statistic parameter mapping of the differences between the “active” and “rest” state images was determined with a *t*-test with statistical significance set at *P* < 0.01. The region of interest (ROI) of activation was processed with MarsBar, a plug-in software for SPM8. This was overlaid to a mean functional image to show the distribution of spinal cord functional activation. The signal intensity changes and the activation voxels in both the axial and sagittal planes were calculated via MarsBar.

### Statistical Analyses

All analyses were performed using SPSS 13.0 software (SPSS Inc., Chicago, IL, USA).

The One-way analysis of variance (ANOVA) was used to analyze age, education, and body mass index (BMI). Sex, smoking status, and alcohol use were tested using a likelihood ratio chi squared test. Differences in signal changes of lumbar spinal cord between the DPN group and the control group were assessed by the non-parametric Mann-Whitney U test. The Spearman correlation test was used to evaluate correlations between the signal changes and the blood biochemical changes. Statistical significance was set at *P* < 0.05.

## Results

**Figure 1:**
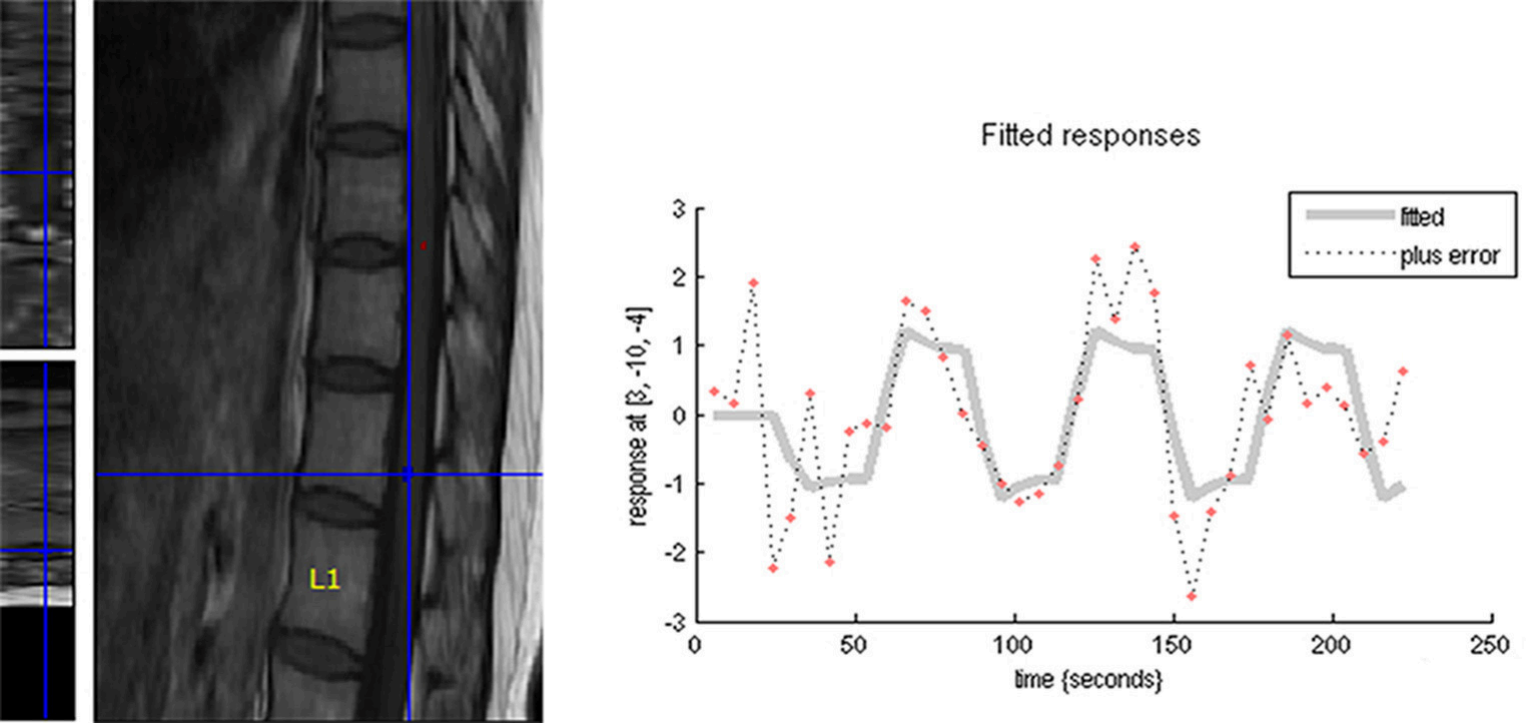
The time-course of the spinal fMRI signal had a good correlation with the rest-active model, indicating that the signal increase was due to the stimulation of the lower extremities, rather than to other causes.

The DPN patients mainly experienced clinical symptoms of sensory dysfunction, numbness, and weakness in lower limbs. The EMG results demonstrated that DPN patients had neuritis of the bilateral lower extremities, with decreased sensory and motor nerve conduction velocity, distal latency, and amplitude. There was no significant difference between the two groups in terms of age, sex, BMI, education, smoking status or alcohol use (*P* > 0.05, Table 1). Lumbar spinal cord activations were found in all controls and DPN patients, though the data of four controls and seven DPN patients were excluded due to excessive movement artifacts, lack of cooperation, or dropout. The time-signal intensity curve of the spinal fMRI signal had a good correlation with the rest-active model, indicating that the signal increase was due to the stimulation of the lower extremities, rather than to other causes (Figure 1).

**Table 1:** Demographic characteristics of healthy volunteers and DPN patients.

	**Control group**	**DPN group**	**Total**	***F/X^**2**^***	***p* value**
Numbers	16	13	29	–	–
Age (years)^a^	42.1 ± 11.1	47.9 ± 8.5	44.7 ± 10.3	2.384	0.134
Education (years)^a^	10.7 ± 4.2	8.5 ± 2.7	9.7 ± 3.7	2.580	0.120
BMI (kg/mm^2^)^a^	24.2 ± 1.3	25.2 ± 1.7	24.6 ± 1.6	2.833	0.104
Sex (M/F)^b^	7/9	8/5	15/14	0.909	0.340
Smoking status (%)^b^	5/16 (31.3%)	6/13 (46.2%)	11/29 (37.9%)	0.677	0.411
Alcohol use (%)^b^	5/16 (31.3%)	5/13 (38.5%)	10/29 (34.5%)	0.165	0.685

a *Age, education, and body mass index (BMI) were analyzed using analysis of variance (ANOVA)*.

b *The sex, smokers, and alcohol users were tested using a chi-squared test. P < 0.05 was considered statistically significant*.

### Lumbar Spinal Activation Characteristics in the Control Group

**Figure 2:**
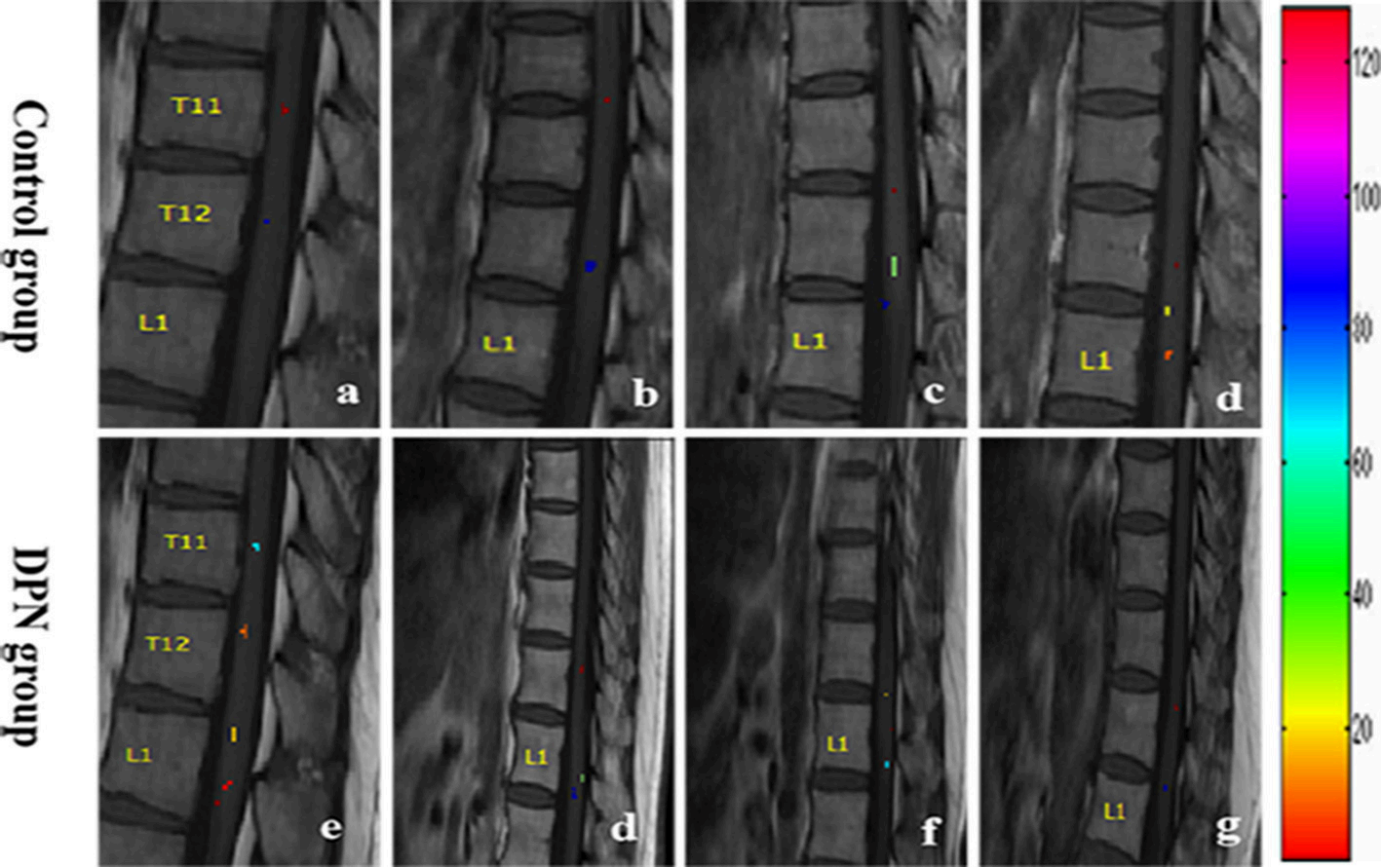
The distribution map of the active signal within a corresponding spinal cord of four different health volunteers **(a–d)** and DPN patients **(e–g)**, respectively. Different colors represented the signal intensity.

**Figure 3:**
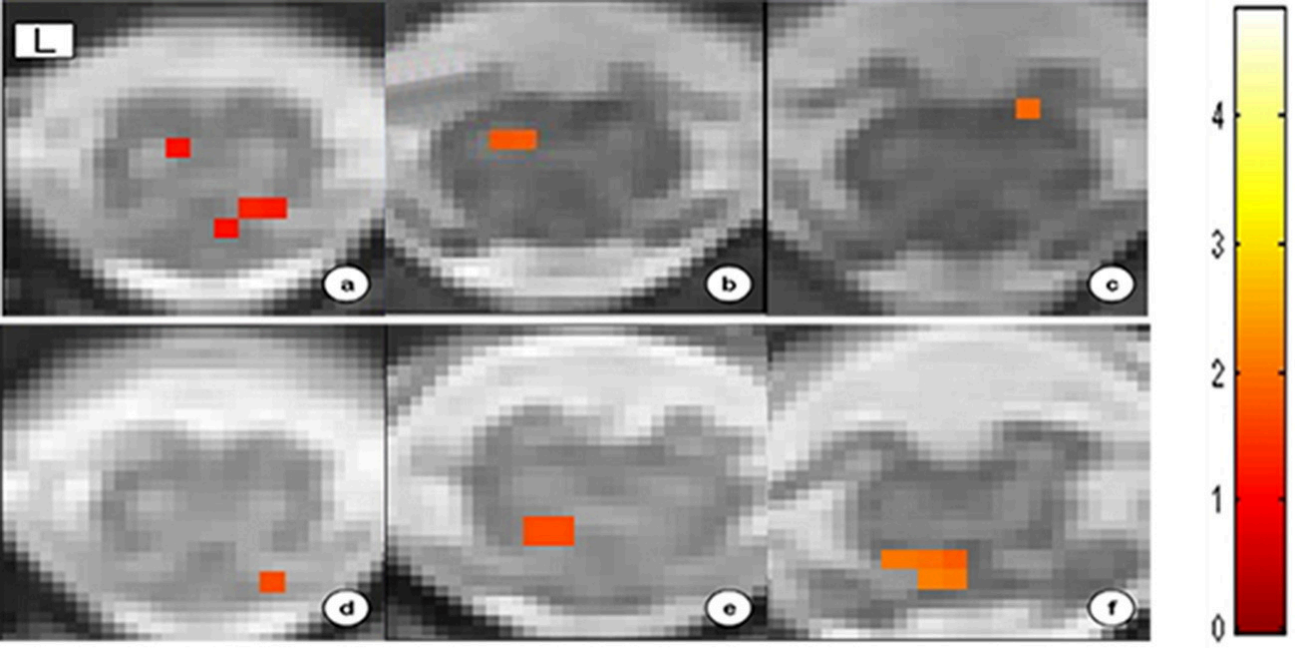
Spatial distribution map of activation signal at the T12 vertebral level within the spinal cord gray matter of six different controls. The ventral **(a–c)** and dorsal **(d–f)** areas represent the motor and sensory neuron activation, respectively.

**Figure 4:**
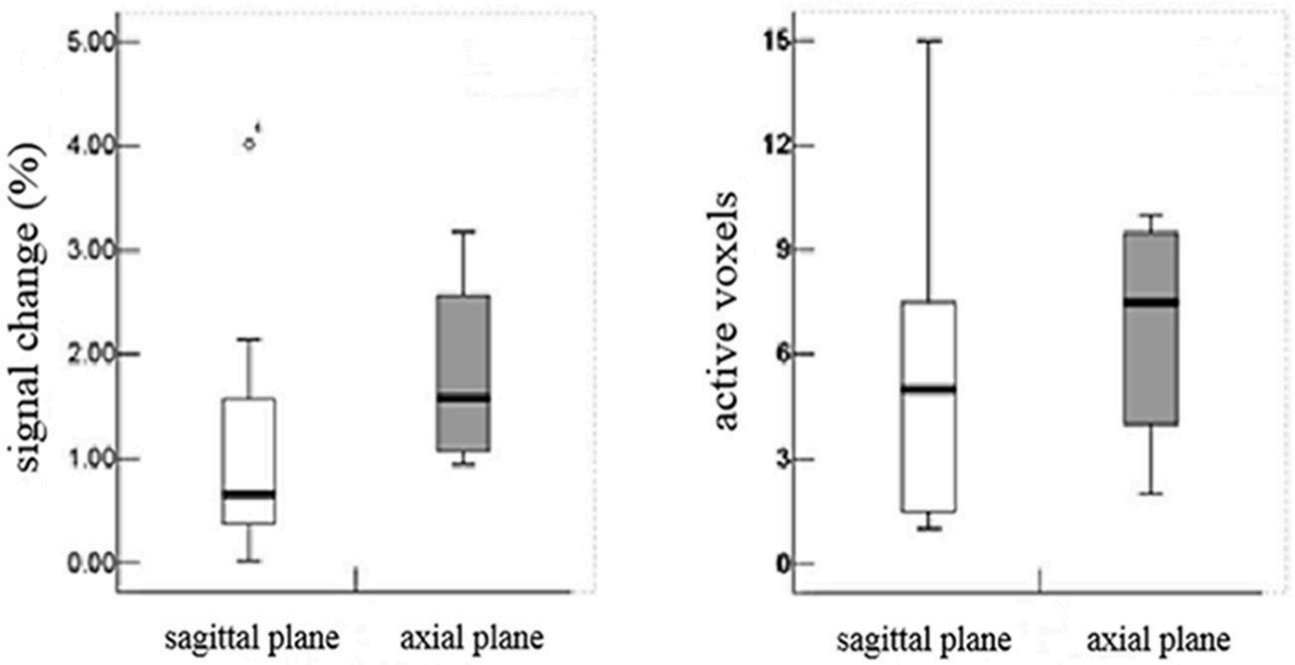
There were no significant differences between the data acquired in axial and the sagittal planes at the T12 vertebral level (*P* > 0.05), indicating that the technique is reliable for functional magnetic resonance imaging of the lumbar spinal cord.

In the sagittal plane, activations were mainly located at the T12 (16/16) vertebral level (Figure 2), with a small number located at the T11 (4/16) and L1 (5/16) levels. The signal intensity change ranged between 0.02–4.02% (95% CI, 0.4–1.8) and the number of activation voxels was 5.25 ± 4.23 (2.5–8.3). In the axial views, active regions were observed in the dorsal horn ipsilateral to the side of stimulation (7/10), and slight activations were found in both the contralateral dorsal horn (5/10) and the bilateral ventral horn (3/10) (Figure 3). The percentage of signal change and the number of activation voxels were 1.82 ± 1.00% and 6.75 ± 3.59, respectively. There were no significant differences between the data acquired in axial and the sagittal planes at the T12 vertebral level (*P* > 0.05), indicating that the technique is reliable for functional magnetic resonance imaging of the lumbar spinal cord (Figure 4). False activations were observed in the CSF and the lower-back muscles adjacent to the vertebral column and were not included in the analysis.

### Functional MRI Differences Between the DPN Group and the Control Group

In the DPN group, the activations of sagittal plane were primarily located at the T12 (10/13) vertebral level, while a small quantity of activity was located at the T11 and L1 vertebral levels. The signal intensity percent change ranged from 0.11 to 8.40% (1.0–5.4) and the number of activation voxels was 5.14 ± 4.50 (2.8–6.5). The percentage of signal changes at T11 and L1 was higher in the DPN group than in the control group (Z = −2.757, *P* < 0.05). The number of activation voxels in the DPN group and the control group showed no significant differences (Z = − 0.077, *P* > 0.05, Table 2).

**Table 2:** Comparison of number of active voxels and signal intensity change percentage between the control and DPN group (Median, 95% CI).

**Group**	**Numbers**	**L1 vertebral level**		**T12 vertebral level**		**Lumbosacral level (T11-L1)**	
		**Voxels**	**Signal change (%)**	**Voxels**	**Signal change (%)**	**Voxels**	**Signal change(%)**
Control	16	4.0 (0.5–8.7)	1.2 (0.1–2.6)	5.0 (2.5–8.3)	0.7 (0.4–1.8)	5.0 (3.0–7.5)	0.8 (0.6–1.8)
DPN	13	5.5 (2.4–10.8)	1.6 (0.9–4.1)	3.5 (2.8–6.5)	2.2 (1.0–5.4)^*^	4.0 (3.1–7.2)	1.6 (1.8–4.1)^*^
*Z*		−0.560	−0.735	−0.156	−2.546	−0.077	−2.575
*P*		0.576	0.462	0.876	0.011	0.938	0.01

The ^*^ represents significant difference at a level of p < 0.05.

### Correlation Between fMRI Activations and Blood Biochemical Indexes

**Figure 5:**
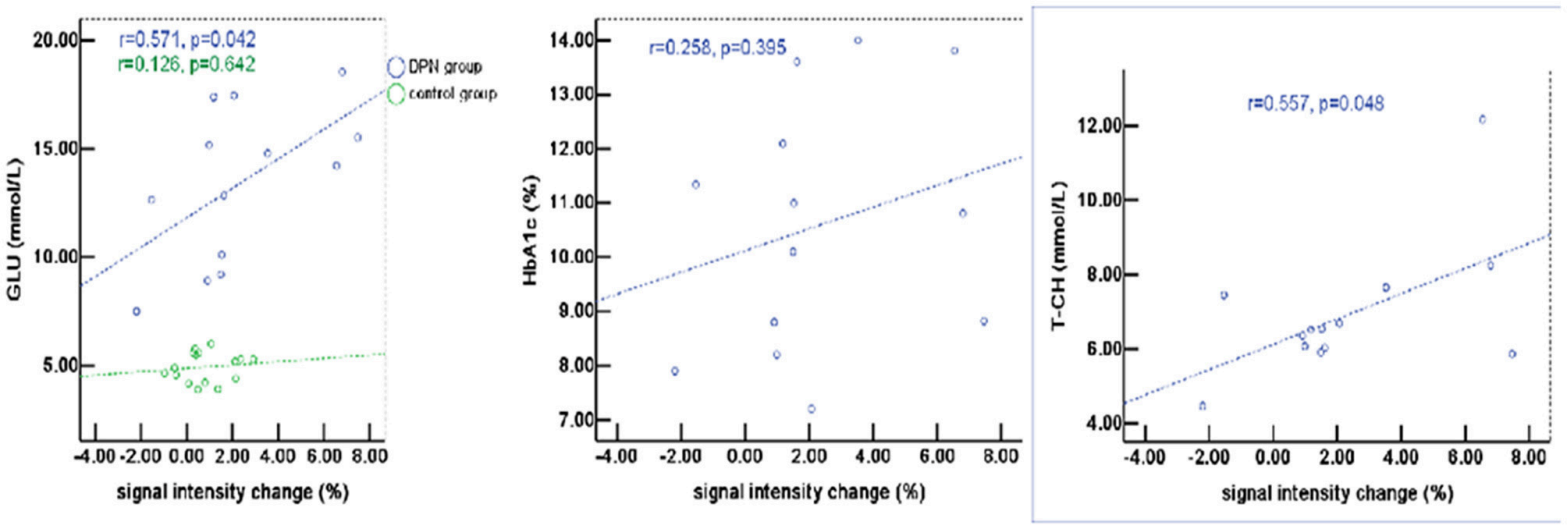
The percentage of signal change in patients with DPN had a positive correlation with blood biochemical changes, particularly with total cholesterol (T-CH) and glucose (GLU) (*P* < 0.05), but no correlation with HbA1c (*P* > 0.05).

**Figure 6:**
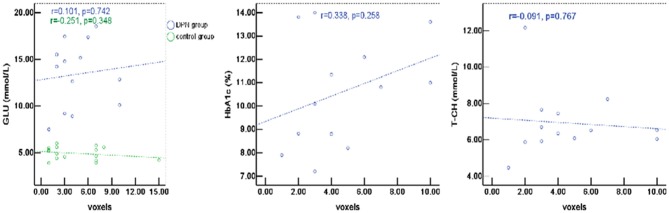
The active voxels of DPN patients had no correlation with blood biochemistry (*P* > 0.05). There was also no correlation between the signal changes and GLU in the control group (*P* > 0.05).

The percentage of signal change in DPN patients had a positive correlation with blood biochemical changes, particularly with total cholesterol (T-CH) and glucose (GLU) (*P* < 0.05), but no correlation with HbA1c (*P* > 0.05, Figure 5). The activation voxels in the DPN group had no correlation with blood biochemistry (*P* > 0.05, Figure 6). There was also no correlation between the signal changes and GLU in the control group (*P* > 0.05).

## Discussion

In this study, we used spinal fMRI to observe the functional activation of the lumbar spinal cord induced by electrical stimulation and investigated the relationship between the signal changes in DPN patients and their blood biochemical changes. SEEP functional activation distribution was observed with electrical stimulation both in healthy controls and DPN patients. There was a greater signal change in DPN patients, with electrical stimulation mainly localized at the T12 vertebral level. Moreover, the signal change in DPN patients was positively correlated with blood biochemical changes, in particular with T-CH and GLU. These results confirmed previous findings in animal studies showing an increase in the signal changes of the BOLD responses after paw stimulation (26–28). In addition, the signal changes observed in this study correlated with the stimulation paradigm on the cord surface and within the abdominal and lower back muscles. These changes outside the spinal canal were the result of task-related motion, changes in the muscle tone, and changes in the blood flow (29, 30). In order to eliminate the apparent intensity changes that arose from systematic shifts in the subject position between periods of rest and activity, image registration was performed on a region containing the spinal canal (over a length of 2–3 vertebrae). The signal located outside the spinal cord was thus excluded from the analysis.

In recent years, human studies of spinal cord fMRI have demonstrated that it is a feasible and repeatable method at a variety of magnetic field strengths: 0.2 T (31), 1.5 T (17, 18), and 3 T (32). As we know, the observed activation in the spinal cord is partly due to BOLD and partly due to SEEP. Since the change in extravascular proton density is field independent, and the BOLD effect can be neglected under conditions of short echo time and low magnetic field, a low field fMRI based on the SEEP effect can be used in the spinal cord. However, spinal cord fMRI has not been widely used clinically at lower field strength (0.2 T), with coarser resolution, or at strong fields (3 T), with higher specific absorption rate (SAR). Thus, in this study, we employed spinal cord fMRI based on the SEEP effect. Finally, the results of our study suggest that the activation obtained correlates well with our knowledge of neural anatomy and that the sensitivity and accuracy of spinal cord fMRI at 1.5 T were similar to those at 3.0 T.

The pattern of the fMRI signal change in the lumbar spinal cord, which correlated with the alternating periods of rest and electrical stimulation of the leg, suggested that the increased signal during stimulation periods was initiated by leg stimulation and not by other events. The number of signal changes detected in the lumbar spinal cord of the control group during stimulation periods was consistent with previous studies that used similar conditions in both human subjects and animal models (13, 20, 29, 33). Regarding activation distribution, activations were primarily observed in the ipsilateral dorsal gray matter (7/10) of the T12 thoracic vertebra. In addition, some activation was found in both the contralateral dorsal horn (5/10) and the bilateral ventral horn (3/10). The areas of activation observed corresponded well with the neural anatomy of the spinal cord, corresponding in most cases to sensory areas. Although electrical stimulation was chosen to provide sensory stimulation, motor stimulation via secondary activation of spinal reflex pathways arc was unavoidable. Of course, motion activity may also be evoked by the body's tiny movement during the electric stimulation. Activation signals located in the contralateral area of the gray matter of the spinal cord may be the result of neuronal activity in the interneuronal ascending pathway.

Several groups have used fMRI to map activity in the human spinal cord, with both positive and negative results reported (34). In our study, we also observed positive and negative activation. Technical issues such as MR artifacts and post-processing data analysis should be considered as the possible mechanisms underlying negative activation, but most importantly such negative activation areas may be caused by decreased or inhibited neural activity due to the physiological structure of neural activity, or by actual decrease of blood flow/volume in these areas. Clearly more research on the mechanisms of negative activation in the spinal cord is needed.

Our study showed that the percentage of signal changes in DPN patients was higher than in controls, a result not consistent with previous animal model studies based on paw stimulation (8). We speculate that in DPN patients it is easy to induce exaggerated activity of primary afferents in the early stages of the disease. Previous studies (8, 9, 11) also demonstrated that the spinal cord and the higher CNS may be involved in sensory information processing, and lead to the behavioral indices of allodynia and hyperalgesia, in which case an increase in fMRI signal would be predicted. The mechanism may involve substance P, a vasodilator, contributing to the BOLD fMRI signal by increasing local blood flow and volume during electrical stimulation (33). The increased synthesis, the axonal transport, and the stimulus-evoked release of substance P from the primary afferents could change the local blood flow, as well as the excitatory stimulation of spinal neurons. Of course, the compensatory effect of nerve injury leading to signal enhancement cannot be excluded. Nevertheless, the percentage of signal changes in DPN patients (8.4%) was lower than in a previous animal study (14.1%), possibly due to the differences in experimental design, stimulation intensity, and data analysis method (8). Therefore, further studies are required, and the precise nature of the increased fMRI signal seen in the spinal cord of DPN patients remain to be clarified. In summary, our data indicates that spinal fMRI is a practical tool, potentially useful for the early detection of diabetic neuropathy.

Although the exact pathogenesis of DPN is still not entirely clear, some research suggests that chronic hyperglycemia causes metabolic disorder, microvascular lesions, oxidative stress, and nervous autoimmune injury, and that peripheral nerve change or necrosis are the result of the combined action of all these factors. It is known that DPN patients usually show some abnormalities in blood biochemical indicators. When insulin secretion and synthesis in diabetes patients are reduced, and/or insulin resistance sets in, alterations of blood glucose metabolism follow, which in turn cause the acceleration of fat decomposition and slow down fat utilization rate, leading to altered lipid metabolism. However, the relationship between the activity of neurons in the lumbar spinal cord and blood biochemistry is unclear. In this pilot study, we analyzed such relationship and found that the percentage of signal change has a positive correlation with GLU and T-CH, consistent with a previous study showing that GLU and T-CH are related risk factors for diabetic neuropathy (35, 36). It is probable that the glucose metabolism disorder caused lipid dysfunction, which then affected neuron activity to some extent. Several studies also found that elevated triglycerides are correlated with myelinated fiber loss (35, 37). These data support the concept that hyperlipidemia may be instrumental in the progression of diabetic neuropathy. There was no correlation between the percentage of signal changes and HbA1c, which could be due to the high level of blood sugar in patients and poor blood sugar control in the last 8–12 weeks. There was no correlation between the activation voxels and the blood biochemical indexes (e.g., glucose, total cholesterol, and Hemoglobin A1c). Therefore, the relationship between signal change and blood biochemical indexes requires further research.

Based on the basic principles and method of SPM8 processing of brain functional imaging, our pilot study demonstrated its application to lumbar spinal cord functional imaging and achieved satisfactory results. However, due to the lack of an appropriate template group analysis for spinal cord was unfeasible. Even so the analysis of individual sessions is important for spinal cord fMRI. SPM8 allowed an analysis of the fMRI signal similar to a recently improved method for spinal functional MRI with large volume coverage of the spinal cord in three dimensions (38). Thus, using SPM8 software to evaluate the activity signal of the lumbar spinal cord it is feasible. A few other factors could be responsible for the variability in the spatial activations observed in this and other studies. These factors can be categorized as sources of artifacts, variability in the physiological response to the stimulation, and limitations in the experimental design (18). Changes in the experimental design could improve the ability to localize activity within the spinal cord. For example, the location of the slice within the lumbosacral enlargement could be refined, and detailed scout images could be used. We chose a slice location (T11-L1 disc) including the L4 and L5 spinal segments, based on typical correlative neuron-anatomy (39), but there was variability between individuals, and additional activation was observed at other slice positions. The observed differences in spinal cord size and quantity of activation could be due to this anatomical variability. In this study, we used averaged images to improve slice registration, due to increased SNR.

Investigating neuronal function rehabilitation will provide a quantitative measurement of rehabilitation progress, and the use of spinal fMRI is appropriate to the task as shown in this study. In addition to being a non-invasive method of detecting neuronal activity, and not limited to a single site of recording, spinal fMRI can be performed on any standard clinical MR scanner without special modifications. Thus, we expect that the development of the spinal cord fMRI technology combined with other MR technologies (40–42) will demonstrate great clinical value for the location of spinal cord function, therapeutic monitoring, and treatment guidance.

## Conclusion

Lumbar spinal cord fMRI, based on the SEEP effect, has been determined as feasible. The repetitive activation distribution was primarily located at the T12 vertebral level. Although the precise physiologic mechanisms and consequences of the increased fMRI signal observed in the spinal cord of diabetes patients remains unclear, our data indicates that fMRI could be used as a tool for the early detection of diabetic neuropathy.

## Data Availability

All datasets generated for this study are included in the manuscript and/or the supplementary files.

## Ethics Statement

This study was carried out in accordance with the recommendations of the human ethics committee of the Second Affiliated Hospital of Shantou University Medical College. All subjects provided written informed consent in accordance with the Declaration of Helsinki.

## Author Contributions

YJ and RW designed the study. YJ, ZS, GL, TN, and TZ performed the research. YJ and ZS analyzed the data. YJ wrote the paper. RW critically revised the manuscript. All the authors approved the final draft.

### Conflict of Interest Statement

The authors declare that the research was conducted in the absence of any commercial or financial relationships that could be construed as a potential conflict of interest.

## Abbreviations

DPN: diabetic peripheral neuropathy
SSFSE: single-shot fast spin echo
EMG: electromyography
SPECT: single photon emission computed tomography
PET: positron emission tomography
MRS: magnetic resonance spectroscopy
fMRI: functional magnetic resonance imaging
BOLD: blood-oxygenation level dependent
SNR: noise ratio
CNR: contrast-to-noise ratio
SSFSE: single-shot fast spin echo
SEEP: signal enhancement from extravascular water protons
TE: echo time
TR: repetition time
ETL: echo train length
NEX: number of excitation
SPM: statistical parametric mapping
GLM: general linear model
ROI: region of interest
ANOVA: analysis of variance
BMI: body mass index
T-CH: total cholesterol
GLU: glucose
HbA1c: hemoglobin A1c.
